# Supporting mental health, wellbeing and study skills in Higher Education: an online intervention system

**DOI:** 10.1186/s13033-018-0233-z

**Published:** 2018-10-06

**Authors:** Alexia Barrable, Marietta Papadatou-Pastou, Patapia Tzotzoli

**Affiliations:** 1 School of Education and Social Work, University of Dundee, Nethergate, Dundee, DD1 4HN UK; 2 School of Education, National and Kapodistrian University of Athens, Athens, Greece; 3 iConcipio Ltd, London, UK; 4 My Psychology Clinic, London, UK

**Keywords:** Mental health, Higher education, Online intervention, Students, Self-help

## Abstract

**Background:**

Dealing with psychological and study skill difficulties can present a challenge for both Higher Education (HE) students, who suffer from them, but also for HE Institutions and their support services. Alternative means of support, such as online interventions, have been identified as cost-effective and efficient ways to provide inclusive support to HE students, removing many of the barriers to help-seeking as well as promoting mental health and wellbeing.

**Case presentation:**

The current case study initially outlines the rigorous approach in the development of one such online intervention system, MePlusMe. It further highlights key features that constitute innovative delivery of evidence-based psychological and educational practice in the areas of mental health, promotion of wellbeing, support of mood and everyday functioning, and study-skills enhancement.

**Conclusions:**

This case study aims to present the innovative features of MePlusMe in relation to current needs and evidence-basis. Finally, it presents future directions in the evaluation, assessment, and evidence of the fitness-for-purpose process.

## Background

Entering Higher Education (HE) is an exciting, yet challenging time. Students have a host of changes to manage, including making new relationships, living apart from friends and family, acquiring new study skills, and learning to function as independent adults [1]. Several studies have indeed shown rising levels of anxiety and depression amongst HE students, compared to pre-university levels [2–4]. Apart from severe mental health issues, mild to moderate psychological difficulties, which occur more frequently, can have a big negative impact on the life of students and often go undetected and unsupported [5]. At the same time, the budget cuts of the HE Institutions (HEI) student support services (SSS) only add to the growing concern about the wellbeing of HE students.

Levels of distress, with anxiety being the most common, rise in the first year of studies and do not fall back down to pre-registration levels during the students’ time at University [2]. Moreover, a substantial decrease in psychological wellbeing (as measured by GP-CORE scores) during the course of a 3-year degree has been reported [2]. A survey conducted in 2013 on behalf of the National Union of Students (NUS) reported that 8% of students identified themselves as “having a mental health problem but not seeking diagnosis” and another 10% as “having been diagnosed with a mental health problem, and I believe it still applies to me” [6]. In terms of reduced wellbeing and experiencing distress, the same report found that 80% of respondents had experienced stress, 70% had experienced lack of energy or motivation, and 55% reported feelings of anxiety. Triggers for such negative feelings can be found mainly in academic responsibilities, such as course deadlines (65%), exam pressures (54%), balancing of studies and other commitments (52%), and grades/academic performance (52%) [6].

The toll of these mental health and study skill challenges can be high. Stress, and its mental health implications, have been found to correlate with negative outcomes, such as poorer relationships and decreased levels of engagement [7]. These same factors were further related to lower graduation rates. Moreover, untreated mild to moderate difficulties can lead to worse outcomes, as well as chronicity [8]. Equally, study skill difficulties can affect students’ ability to benefit fully from HE and reach their full academic potential [9]. On the other hand, wellbeing, seems to have a positive effect on educational achievement and flourishing [4, 10].

The toll on HEI is also substantial. Not only do universities have a duty of care towards their students, but HE has increasingly become a consumer-driven market and student satisfaction is important in all areas, including students’ living and social environments, putting further pressure on HEI to provide a holistic experience [11]. Recent increases in UK HEI fees only add to student expectations for a memorable “student experience”. This, in turn, can influence the ratings, intake, and income of the HEI.

As demand for support has grown steadily [12], both HEI and external agencies (GP practices, NHS mental health services, and voluntary organisations) have faced increasing pressure on resources [13]. Funding, which is sourced from the Disabled Students’ Association (DSA) as well as through HEI’s central funds, has been difficult to allocate. It is central funding, mainly from fees, that is allocated for more general provision, including disability services and counselling [13]. On-campus SSS are struggling to meet this demand [14], resulting in long waiting lists and lengthy waiting times [15]. Such waiting times can sometimes be as long as 9 weeks, meaning students are left without access to support for the length of an academic term on occasion [16]. Alternative routes, such as seeking help through private routes, can be a possibility, but come at a high cost, one that most students do not have the budget for. Furthermore, the SSS are left with no choice but to prioritise cases, preferentially allocate resources to students with severe difficulties. This inevitable practice leaves students who face mild to moderate difficulties -the majority of the student population- with no support.

Research on help-seeking behaviours suggests that many students who suffer distress often don’t reach out at all [17]. Fear of being stigmatised has been identified as one of the reasons for this breakdown between needing and seeking help [18], resulting in calls for interventions that reduce stigma [7]. Universal online intervention programmes have been identified as one possible way to engage students in need of mental health and wellness support, who are unlikely to seek formal help [19, 20]. The Royal College of Psychiatrists also suggests web-based interactive cognitive behavioural therapy (CBT) programmes to support students [15]. In an attempt to meet this increased need for support to students with mild and/or moderate difficulties, an online support system designed specifically for HE students was developed. Its development and rationale will be the focus of this case study, alongside the benefits for both students and HEI. The innovation aspect of this online support system, in providing something that is not currently available, will also be highlighted. The extent to which the system described here constitutes an innovation, lies largely within the combination of features that are outlined on Table 1, but mainly in its bottom-up design (i.e., how it works). This point is further developed at the end of this article.

**Table 1:** Features, evidence base and benefits of the system

MePlusMe feature	Theory/evidence	Benefit for user	Benefit for HEI
Online access	Effectiveness of online therapies [19]	Ease and flexibility of access Anonymity (removal of stigma)	Freeing up resources in SSS for students with more severe difficulties
Visual appearance, language and layout designed specifically for students	Proof-of-concept study—end-user feedback [21–23]	High engagement levels Inclusive A system that is ‘fit-for-purpose’ and highly usable	Marketing their different support services tailored for students
Ability to personalise page (both for user and HEI)	Personalising a product can strengthen emotional bond [23]	Strengthens emotional bond to system. Student takes ownership	Marketing possibilities (white labelling)
Use of multimedia (animated videos, etc.)	Cognitive Theory of Multimedia Learning [24]	Enhanced learning environment	Claiming variability in the different offerings for students’ support
Assessment measure (Questionnaire) developed from validated clinical tools	HADS [25] GAD-7 [26] PHQ-9 [27] MINI [28]	Accurate identification of needs. Best-fit intervention	Untrained staff (e.g., personal tutors) can use the Questionnaire as a first-line symptom identification tool
Evidence-based techniques used (e.g., normalisation, promoting wellbeing, problem solving, study skills)	CBT [29], behavioural activation [30] cognitive defusion [31–33]	High effectiveness in addressing symptoms, enhancing general wellbeing and increase academic self-efficacy	Fit-for purpose
Availability of granular statistics analysis	Data granularity allows for a better analysis of data [34]	Indirectly, as HEI allocate resources better and are able to adequately support students	Improvement of resource allocation. Stakeholder accountability Improvement of reputation and profitability Branding opportunity to be labelled as the “caring” university

## Development and evaluation

MePlusMe is an online, multimedia intervention designed to meet the needs of students with mild to moderate psychological and study skills difficulties. The system offers assistance in supporting mood and wellbeing and in developing effective study skills. A rigorous approach to the development MePlusMe has been adopted. An initial consultation to help begin conceptualising the development of the system and to tap on the knowledge and experience of mental health providers within four HEIs; London School of Economics, University College London, King’s College London, and Kingston University. The questions focused on students’ most common difficulties at university, current practices and challenges within institutions, and inquired about the need for and design of a potential online solution. At the same time, HE students (*n* = 61) were surveyed, using an online survey tool (to better understand their needs at university, their potential engagement with an online system, and their preferences with regards to features, functions, and style of such a system [21].

Furthermore, in a proof-of-concept study [22] 873 students from five UK HEI (King’s College London, University of Warwick, University of Edinburgh, Bournemouth University, and University of Roehampton) took the online surveys and their responses confirmed suitability of the system and that it addresses students’ needs in HE. These responses informed further development on the visual aesthetics, language, and functionalities of the system.

### Fitness for purpose

There are two different routes into the available content, either through a ‘symptoms route’, which comprises of a questionnaire that assesses the individual needs of the user and provides a custom-built package of multimedia content that aims to address these issues, or through the ‘Library’, which relies on user selection of content, depending on their needs at the time. The content is then available for users to access at their leisure.

The ability to choose between the two available routes when using the system gives students control of how to access help depending on their situation at any given instance. Students who can identify their symptoms but don’t know what help they need can go through the Questionnaire route, while students who know what help they are after can go directly to the Library. The flexibility of this approach, which is integral to the system, aims to help students feel empowered by choosing how to get the help they need.

Furthermore, the Questionnaire has been meticulously developed through the adaptation of established clinical instruments to ensure effective differentiation between different categories of symptoms. Those instruments were the Hospital Anxiety and Depression Scale (HADS) [25], the 7-item Generalised Anxiety Disorder Scale (GAD-7) [26], the Patient Health Questionnaire (PHQ-9) [27] and a formal interview (Mini International Neuropsychiatric Interview [28]. The adapted Questionnaire was piloted on (*n *= 491) students for its ability to provide an initial filtering process and identification of presenting difficulties [35]. The main aim is to provide effective differentiation between three categories of presenting problems, namely predominantly anxiety, predominantly depression or a mix of both. However instead of a diagnosis, MePlusMe makes use of symptoms and links clusters of symptoms with specific evidence-based psychological techniques. Therefore, once the Questionnaire is completed the user is presented with a tailor-made package based on the identified needs. In other words, this bottom-up design allows students to visit the system as many times as they need during their studies and get immediately bespoke advice to address the specific difficulties they face each time. This is effectively the most innovative element of MePlusMe.

The system offers optional motivational reminders, provided as the default option of MyPlan, in the form of emails aim to encourage users to return to the system regularly. A self-rating system is used to monitor progress for 8 weeks and provides feedback to students at specific intervals in the form of a graph. At week 8 the students also receive a stamp (i.e., badge) with the message “I did it!” on their completed graph to reward their effort, perseverance, and achievement.

A within-the-system social network is provided for users in the form of a Thoughtwall—a space where students can post their thoughts and feelings whilst using the system; students can also share their graph once they completed working on a particular topic at the end of the 8 week period. This space is monitored by expert online moderators and thoughts are going live within 24 h. Engagement of users is encouraged, with the option of (a) ‘sharing’ their own posts, including a feature that allows users to post them onto other social media, like Facebook and Twitter and (b) ‘liking’ other users’ thoughts. Issues of anonymity have been addressed, by the ‘thoughts’ appearing under the username that was originally specified by users during registration. It is the users’ choice if they decide to share their thoughts under their username from their social media account [35].

MePlusMe, uses a host of evidence-based techniques to address psychological difficulties. Evidence on the efficacy of online interventions [36] and findings on the effectiveness of guided self-help as a mean of delivery compared to face-to-face [37], informed both the design and the content of the system. In particular, cognitive behavioural therapy (CBT) and Problem Solving Therapy (PST) are the core behind the design of this online intervention system. As a result, techniques that have been shown to be successful in treating symptoms of anxiety, such as a Worry Decision Tree [38], and of depression, such as Behavioural Activation [31] have been incorporated into the design. Cognitive Defusion, a technique that can be used to deal with negative self-talk or thoughts [31–33], is also part of MePlusMe’s toolkit. Techniques that can help with study skills difficulties (e.g., time management, staying motivated, and memory techniques) were also chosen.

All the above content is delivered through several channels. Animated videos, as well as short text have been utilised to present different materials within MePlusMe. This has been built according to the Cognitive Theory of Multimedia Learning [39] and relies on the assumptions that the active process of learning, which involves filtering, selecting and organising information, can be facilitated through the use of different channels. This makes the assimilation of information easier for the user, and the learning process more effective.

The content, language, visuals, sounds and overall the feel, layout, and functionality of MePlusMe have been heavily influenced by students’ feedback following Goozée et al.’s survey [21] and a proof-of-concept study [23]. Examples of this outcome are: the specific difficulties (symptoms of anxiety and depression) targeted to be addressed via the chosen techniques, the way questions were phrased in the questionnaire and the language used in the videos’ scripts, the colours and graphics throughout the system including the voiceover and the male and female character featured in the videos as well as features such as the blackout button that allows students to “escape” quickly to another website. Furthermore, the system was carefully developed in order to consistently avoid using pathologising language and diagnostic terms and focuses instead on describing the symptoms and experiences targeted.

The ability for users to personalise the platform has also been added to the system. For example, students can choose their own pictures hanging on the “wall” or their own scenery from the “window”. Personalised products have been found to elicit greater emotional investment [23, 40], thus this feature could increase the users’ ‘emotional bonding’ to the system. In that way it aims to promote repeated use and better engagement.

### Target group and filtering processes

MePlusMe has been designed specifically to address the needs of HE students. The system targets needs that call for psychological and/or study skill support and which are relevant to the biggest subset of the student population that has largely been ignored thus far: individuals with subclinical symptoms of depression and anxiety that would perhaps ‘fall through the cracks’ of traditional support systems, or face long waiting times when seeking treatment. MePlusMe pitches itself to this big bulk of students who at some point during their studies will experience mild to moderate difficulties and has been designed with this specific target group in mind. However, students with no symptoms, but a desire to improve their personal effectiveness, may also feel attracted to use the system given its unassuming design that emphasises solutions and avoids stigmatisation. The system is welcoming and open to students who wish to use it for such purposes.

Several filtering processes are in place, to ensure that students who need added levels of support can be directed to the right services. A highly visible “In case of emergency” button (i.e., panic button) has been placed in a prominent spot in the first screen a user encounters when enters MePlusMe’s Home page. This is to be used in case of immediate and pressing difficulties felt by the user, and provides immediate access to alternative routes, such as mainstream mental health helplines or contact with the HEI’s own SSS. The use of such panic buttons is widespread in online and mobile technologies that address mental health issues [41].

The second filtering system in place at initial login and it is a host of screening statements, which the user is asked to agree or disagree with, such as “You have taken risks to the point where it regularly affects your health and/or disrupts your daily life”. Every statement has an underlined word that is further explained with examples. If a user relates to these statements then they are provided with further guidance, which consists of alternative routes of support. These can include going to their primary healthcare provider or seeking support within the University SSS. Users who at the time might not relate to these statements can proceed using the system, but are again presented with these statements every 3 months. The aim of this is to have a robust and consistent monitoring system in place, in case users’ difficulties escalate. In those cases a user is again presented with information on how to access appropriate help.

Another filter put in place is the completion of the Visual Analogue Scale (VAS) by the student every few weeks from commencement of use of the system (week 2, 4, and 8). The system is designed so that if two consecutive scores are recorded as low on the system, the user receives a message in their personal space within MePlusMe (MyMessages) and is advised to seek further support via the SSS or through primary care. Frequent monitoring is a vital feature of the system. It ensures that students with severe difficulties are being directed to more appropriate sources of support.

Finally, if a user, whilst answering the Questionnaire, does not relate to any of the presented symptoms, they are advised to look through Library content in case they find a technique that could be useful to them. They are further advised to seek alternative support services that could be more suited to their needs at the time. This path acts as another filter to identify students with difficulties that can be best addressed by other, more appropriate, services.

## Benefits for students

### Removing barriers to help-seeking

MePlusMe’s overall layout, language, and design has been developed in a way that is culture- and specific-learning-difficulties-sensitive and that normalises students’ experiences. As such it removes a lot of the barriers to help-seeking for young adults [42–44], thus improving access to support, wherever the latter is needed. It can attract students from different ethnic backgrounds; it is accessible to students averse to reading long texts, and it is also inviting to students with no symptoms who wish to use the system as a source of information and skill development. This inclusiveness removes any potential perceived stigma which often interferes with help-seeking [18], while at the same time it brands the system as more of a source of learning and improving one’s own self efficacy. The panic button, the ongoing screening process, the Questionnaire and the self-monitoring feature ensure that users who have severe mental health difficulties are receiving more appropriate information with regards to where and how to seek help, including the HEI’s SSS, primary care providers, or other professionals.

### Promoting wellbeing

Much of research and public policy remains focused on mental illness and other pathologies, often disregarding the positive aspects of mental health and wellbeing. However, mental and psychological wellbeing are both desirable outcomes in themselves, as well as linked to a host of other positive outcomes, such as better physical health [45], improved performance at work or school, lower levels of absenteeism, and more satisfying and successful relationships [46–48]. In essence, optimal wellbeing is linked to actively flourishing in all aspects of life [49].

Promoting mental health and wellbeing is very much at the centre of what MePlusMe aims to deliver. It is based on a continuum model of mental health [50] and advocates a normalising rationale of symptoms emergence for students. In addition, it promotes wellbeing through the active recommendation of healthier habits linked to physical wellbeing, such as better sleep, physical exercise and healthy eating, as well as activities that have been associated with better psychological and subjective wellbeing. Practising gratitude, socialising, and helping others have all been found to increase wellbeing in non-clinical samples [51–53] and have been included in MePlusMe’s design.

Furthermore, MePlusMe aims to promote self-efficacy, linked to wellbeing [54], through offering students a sense of control in dealing with their difficulties. Academic self-efficacy, promoted through the system’s study related skills, has been found to play an important role in both maintaining positive emotion, as well as better academic performance [55]. Moreover, by promoting greater overall self-efficacy, academic performance could be improved [56]. Finally, smoother adjustment into university life and higher commitment of remaining in HE are also correlated with academic self-efficacy [57].

The study skills training is supported by MePlusMe through a range of techniques, such as keeping motivation levels high, time management, as well as advice on revision and learning different memory techniques. Although usually study skills are accumulated through practice, trial and error, there are certain techniques, which have been incorporated into MePlusMe, that can accelerate the process of gaining these skills and help students deal with the academic challenges they face more effectively [58].

### General benefits from online interventions

Aside from the above benefits, which are specific to this system, there are certain advantages that most, if not all, online systems share, such as 24/7 availability, anonymity, access from a variety of devices (i.e., smartphones, tablets, computers, and laptops) and from the comfort of the users’ personal space and chosen location [41]. Having online access to the assessment of one’s own needs and an intervention solution removes several of the barriers to seeking help in young adults [59]. For example, a major concern of young adults accessing mental health support lies in confidentiality and trust, as well as stigma [43]. As MePlusMe provides anonymity and does not include any face-to-face contact, it could diminish young people’s concerns over confidentiality or stigmatisation.

Also, the system’s model avoids the “one size fits all” common approach and as a result offers students the autonomy on how to use it that matches their style and schedule, as well as offering them access to best-fit interventions for their needs, as they arise. As a result, this empowers them, reinforces their self-efficacy, improves their confidence and motivation to change as well as promote their mental wellbeing.

### Benefits for HEI

Looking at the benefits for HEI, the system indirectly frees up resources of face-to-face services for those students who have a greater need for them. It can also be seen as complementary to SSS as it can also be used while a student is awaiting referral through over-subscribed services. Additionally, it could potentially be used as a back-up support system once acute problems have been dealt with by SSS, facilitating self-monitoring to those who need it. Furthermore, untrained staff (e.g., personal tutors) can use the Questionnaire as a first-line symptom identification tool. Therefore, MePlusMe can help HEIs meet more effectively their pastoral role to the students and brand them as “the caring university”.

Finally, HEIs can also benefit by making use of the granular statistical analysis of the data collected via MePlusMe as well as the combination of the latter results with findings from the clinical measures and the feedback form. These data can offer insights on monitoring overall use, accessing data on student mental health and academic competence, improve demand management, facilitate referral and identify how existing services could be further resourced and optimised. Such analytics can be further used to improve accountability to stakeholders, on budgeting and resources. Similar online interventions for mental health have been found to be cost-effective [60] thus these data can also lead to a potential better allocation of resources within HEI.

### MePlusMe—the innovative aspects

Innovation in mental health services, especially in relation to the use of technologies, has been called for in the past [61]. The use of online evidence-based programmes to provide an alternative to face-to-face services has been seen as a welcome innovation in mental health support provision [15] and has become a focus for current mental health-care research [62]. Innovation, within the field of mental health services should aim to meet the needs of the end-user, by providing solutions that are not simply “more of the same” [62]. In this context, MePlusMe constitutes such an innovation and this section will aim to highlight the ways in which it does, in relation to other currently available web-based support systems designed to be used by students.

A 2017 review of web-based mental health support systems identified six such systems that were specifically designed to address the needs of the HE student population [41]. Of the six, two have been designed and used in Ireland and Australia and four of them have been designed for the UK market. Of those four, MePlusMe is the only system designed to address both psychological and study skill difficulties whilst the others focus solely on psychological issues. Moreover, it is unique in relation to the other available systems in that its mode of delivery is based on the use of multimedia whilst the other systems’ content is heavily based on text. As described above, MePlusMe has been designed according to the Cognitive Theory of Multimedia Learning [39], and aims to provide a more effective learning process for the end-user. MePlusMe is also the only system that has been influenced directly by students’ feedback and as such it has been developing hand-in-hand with the end-users [21–23]. Finally, and most importantly, whilst other systems offer the same pre-made packages or modules to all users for specific conditions, MePlusMe is the only system that after every use it offers students a tailored solution to their needs because it addresses symptoms as opposed to diagnosed conditions and links them with specific psychological techniques.

To summarise, it is the combination of its various features that constitutes MePlusMe an innovation in the world of web-based support systems. As analysed in detail in this article, these are (1) The targeted design for HE students as the end-user, (2) the use of multimedia for delivery of materials, (3) the inclusion of study-skills difficulties in the repertoire of issues to be addressed, but most importantly, (4) it’s bottom up design that offers personalised advice after every use. In this way, MePlusMe is innovative and comes to meet the growing need for support towards HE students in a period of transition.

## Conclusion and future directions

The current case study has attempted to describe MePlusMe, a web-based support system for students, its development and the benefits it offers to both students and HEIs. MePlusMe is innovative in several ways. It addresses previously unmet needs, namely subclinical problems alongside general wellbeing and study skills. The former are problems that often go unnoticed, while the latter two have not been at the centre of traditional SSS, which focus largely on treating acute and chronic mental health issues, thus prioritising according to the “severity of the distress [and] disability” as well as on the “impact on academic progress” (Royal College of Psychiatrists, 2011, p. 20 [41]. Additionally, it does so in a unique way; it avoids one size fits all solutions and offers personalised interventions by creating different packages with techniques every time students use the system tailored around their needs at the time.

Moreover, it comes at a time when the prevalence of psychological and study skill difficulties in HE students, paired with decreasing government spending, presents a challenge in meeting students’ needs, especially with regard to mild and moderate mental health difficulties, or difficulties relating to study skills. In this climate, MePlusMe, comes to meet many of the above needs, providing easy, stigma-free access to psychoeducation and support. Its role can be seen as complementary to traditional face-to-face support services, meeting the needs of students who often “fall through the cracks” of SSS, as their difficulties are sub-clinical.

It has been shown that the inception and development of the system are based on the best current available evidence. In due course, further empirical studies should contribute to the establishment of a solid evidence-basis behind the use of MePlusMe to support the wellbeing and study skills of students leading to a wide-scale incorporation of the system within HEI SSS. A pilot study was recently completed at the University of West London that aimed to assess the likeability of MePlusMe’s design and the feasibility of its content. Next, a larger feasibility study will take place before a randomised controlled trial is planned, with a number of different universities, to compare other interventions, including treatment-as-usual or placebo with MePlusMe and establish its relative effectiveness.
